# BRAF Inhibitors in Thyroid Cancer: Clinical Impact, Mechanisms of Resistance and Future Perspectives

**DOI:** 10.3390/cancers11091388

**Published:** 2019-09-18

**Authors:** Fabiana Crispo, Tiziana Notarangelo, Michele Pietrafesa, Giacomo Lettini, Giovanni Storto, Alessandro Sgambato, Francesca Maddalena, Matteo Landriscina

**Affiliations:** 1 Laboratory of Pre-Clinical and Translational Research, Centro di Riferimento Oncologico della Basilicata, Rionero in Vulture, 85100 Potenza, Italy; fabiana.crispo@crob.it (F.C.); tiziana.notarangelo@crob.it (T.N.); michele.pietrafesa@crob.it (M.P.); giacomo.lettini@crob.it (G.L.); alessandro.sgambato@crob.it (A.S.); 2 Nuclear Medicine Unit, IRCCS, Centro di Riferimento Oncologico della Basilicata, Rionero in Vulture, 85100 Potenza, Italy; giosto24@hotmail.com; 3 Medical Oncology Unit, Department of Medical and Surgical Sciences, University of Foggia, 71121 Foggia, Italy

**Keywords:** BRAF mutation, thyroid cancer, BRAF inhibitors, mechanism of resistance

## Abstract

The Kirsten rat sarcoma viral oncogene homolog (RAS)/v-raf-1 murine leukemia viral oncogene homolog 1 (RAF)/mitogen-activated protein kinase 1 (MAPK) signaling cascade is the most important oncogenic pathway in human cancers. Tumors leading mutations in the gene encoding for v-raf murine sarcoma viral oncogene homolog B (BRAF) serine-threonine kinase are reliant on the MAPK signaling pathway for their growth and survival. Indeed, the constitutive activation of MAPK pathway results in continuous stimulation of cell proliferation, enhancement of the apoptotic threshold and induction of a migratory and metastatic phenotype. In a clinical perspective, this scenario opens to the possibility of targeting BRAF pathway for therapy. Thyroid carcinomas (TCs) bearing BRAF mutations represent approximately 29–83% of human thyroid malignancies and, differently from melanomas, are less sensitive to BRAF inhibitors and develop primary or acquired resistance due to mutational events or activation of alternative signaling pathways able to reactivate ERK signaling. In this review, we provide an overview on the current knowledge concerning the mechanisms leading to resistance to BRAF inhibitors in human thyroid carcinomas and discuss the potential therapeutic strategies, including combinations of BRAF inhibitors with other targeted agents, which might be employed to overcome drug resistance and potentiate the activity of single agent BRAF inhibitors.

## 1. Introduction: Epidemiology, Classification and Molecular Features of Thyroid Cancer

Thyroid cancer (TC) is the most common endocrine malignancy, representing approximately 2.1% of all newly diagnosed tumors in the world. In the last decades, the annual incidence has almost tripled and the same trend can be observed in prevalence rates. TC occurs at any age, with higher incidence in individuals aged 25–60 years; however, women have a fourfold higher incidence than men. The survival rate is very high, with a five-year survival rate exceeding 90% for differentiated tumor subtypes [1]. 

The vast majority of TCs (95% of cases) derives from thyroid follicular epithelial cells, whereas the remaining 3–5% originates from parafollicular C cells [2]. TCs of follicular origin can be histologically classified into different histotypes [3]: (i) papillary thyroid carcinoma (PTC), the most common type of TC (75% of cases), frequently asymptomatic and characterized by a good prognosis; (ii) follicular thyroid carcinoma (FTC) (15% of cases), the second most common histotype with a worse prognosis and a greater incidence in geographic areas with an inadequate iodine dietary intake; (iii) anaplastic thyroid carcinoma (ATC), an undifferentiated form of TC, with an incidence lower than 1% and characterized by aggressiveness and rapid metastatization. Medullary thyroid cancer (MTC), originating with parafollicular C cells, is a neuroendocrine tumor that secretes high levels of calcitonin, generally sporadic, even though 20–30% of cases are familial due to autosomal dominant germline mutations in Rearranged during Transfection (*RET*) proto-oncogene [4].

Follicular-derived thyroid cancers can be further classified depending on cell differentiation, where the term “differentiation” refers to the maintenance of cellular functions that are typical of normal thyroid follicular cells, first of all the thyroid stimulating hormone (TSH) dependency, which increases the probability of a beneficial response to radioiodine (RAI) therapy. Well-differentiated thyroid cancers (WDTCs) are the most prevalent type of TC (85–90% of all cases) and include papillary and follicular tumors. WDTCs have an asymptomatic clinical evolution and an excellent prognosis with low morbidity and mortality [5]. ATCs are characterized by lack of cell markers of thyroid differentiation and this is due to the de novo appearance of aggressive tumors devoid of typical thyroid traits or to the progressive dedifferentiation of initially well-differentiated tumors. During the dedifferentiation process, ATCs lose the TSH dependency and the ability to express the sodium-iodide symporter upon TSH stimulation, causing the loss of response to RAI therapy. Among all TCs, anaplastic subtypes show the most aggressive clinical behavior and the poorest prognosis [6]. Poorly-differentiated thyroid carcinomas (PDTCs) represent the bridge between WDTCs and ATCs. Recognized in 2004 by the World Health Organization Classification of Endocrine Tumors as a distinct entity [7], PDTCs are characterized by a singular histologic architecture and the absence of the conventional nuclear patterns of papillary thyroid carcinomas (PTCs), beyond some distinctive features, such as convoluted nuclei, mitotic activity or necrosis [8]. Due to their poor differentiation and greater aggressiveness, generally PDTCs exhibit increased local growth and distant metastatization and a worse overall survival (OS) with respect to WDTCs [8]. 

In a molecular perspective, it is known that genetic anomalies of specific signaling pathways are key factors in thyroid tumorigenesis [9,10,11,12]. Indeed, the constitutive activation of MAPK and phosphatidylinositol-4,5-bisphosphate 3-kinase catalytic subunit alpha (PI3K)/v-akt murine thymoma viral oncogene homolog 1 (AKT) signaling cascades due to mutational events represents crucial molecular steps in thyroid carcinogenesis. In particular two main mechanisms, mutually exclusive, are involved in the hyperactivation of MAPK and PI3K/AKT pathways: (i) activating mutations/rearrangements in proto-oncogenes, such as rat sarcoma viral oncogene homolog (*RAS*), v-raf murine sarcoma viral oncogene homolog B1 (*BRAF*) or *RET/PTC*; (ii) recessive mutations in tumor suppressor genes, which cause loss of function [10,11]. Indeed, the abnormal activation of the MAPK cascade, due to mutations and/or rearrangements in *RET*, *RAS* and *BRAF* genes, characterizes approximately 70% of PTC cases [9], and, among these, the rearrangements of the *RET* gene that lead to the formation of a constitutively active fusion protein occur in 10–20% of cases [11]. 

Numerous research projects have demonstrated that *RAS* mutations represent an early event in the adenoma stage and may drive malignant transformation. Additionally, evidences suggest that RAS mutations may sustain WDTC dedifferentiation into PDTC and ATC [13]. However, the prognostic value of RAS mutations in predicting aggressiveness, recurrence and response to therapies is still a matter of debate. The *RET/PTC* rearrangement causes the fusion of the 3′ portion of the *RET* gene, which encodes for a tyrosine kinase receptor highly expressed in C parafollicular cells, with the 5′ portion of histone 4 (*H4*) or nuclear receptor coactivator 4 (*NCOA4*) genes. RET/PTC rearrangements are common in radiation-induced and sporadic young tumors, where the aberrant expression of the *RET/PTC* fusion gene constitutively activates RAS/RAF/ERK pathway, promoting follicular malignant transformation [14]. BRAF mutations occur in approximately 29–83% human TCs and the V600E mutation is the most frequent [15]. Generally, these mutations are responsible for an aggressive biological and clinical behavior, loss of thyroid differentiation with reduced response to RAI therapy and poorer OS [16]. In recent years, several specific BRAF inhibitors (BRAFi) were developed and entered in clinical practice [17], mostly in melanoma. In the context of TCs, the clinical activity of these targeted agents has been object of intense investigation with conflicting evidences. While Food and Drug Administration (FDA) and European Medical Agency (EMA) approved these agents in BRAF-mutated TCs, drug resistance represents a major issue for their clinical use and a limitation for patient outcome. This review will discuss the molecular mechanisms responsible for resistance to BRAFi in TCs and the therapeutic strategies with combination of BRAFi with other targeted agents to overcome drug resistance. 

## 2. BRAF Signaling in Cancer

*BRAF* gene encodes for cytoplasmic protein belonging to the RAF serine/threonine kinase family [18]. Structurally, BRAF protein is composed of three highly conserved regions: (i) the RAS binding site, CR1; (ii) the regulatory domain, CR2; (iii) the catalytic domain, CR3 with kinase activity involved in ATP and substrate protein binding. CR1 and CR2 are located in the N-terminal region, whereas CR3 is located in the C-terminal region and contains the activation segment and the regulatory region, fundamental for BRAF activation [19]. There are three RAF isoforms: ARAF, CRAF and BRAF. Among these, the BRAF kinase has the strongest basal activity and plays a key role in the regulation of the RAS/RAF/MEK/ERK signaling pathway, which is highly conserved in eukaryotes, is responsible for nuclear responses to extracellular environmental stimuli [19] and controls essential cellular processes such as embryogenesis, cell differentiation, proliferation and migration, growth and survival [20,21].

Genetic alterations and/or dysfunctions in regulatory mechanisms of RAS/RAF/MEK/ERK pathway play important roles in the development of cancer [22]. Several studies showed that the main activating mutations of RAS/RAF/MEK/ERK transduction pathway occur in the following genes: (i) V-Ki-ras2 Kirsten RAS (*KRAS*); (ii) neuroblastoma RAS (*NRAS*); (iii) Harvey RAS (*HRAS*) and (iv) *BRAF* proto-oncogenes. 

More than 40 different mutations in *BRAF* gene have been identified in human tumors, mainly localized in the CR3 region. *BRAF* mutations have a very high frequency in hairy cell leukemia (80–100%) [23], melanoma (30–70%) [24], papillary thyroid tumors (36–83%) [16], type I ovarian (30%) [25] and colorectal cancer (10%) [26]. Instead, a low prevalence of BRAF mutations was found in non-small cell lung tumors (<5%) [27].

Ninety percent of BRAF mutations consist of a nucleotide substitution of a thymine with an adenine at position 1799 in exon 15. This missense mutation causes the replacement of a valine (V) with a glutamic acid (E) at amino acid residue 600 (BRAF^V600E^) [28]. Other BRAF point mutations [28], as well as deletions and gene fusions [29,30], have been extensively described by several studies, even though their prognostic/predictive role is still matter of debate [29,30,31,32]. Instead, the BRAF^V600E^ mutation is the most clinically relevant, because of its involvement in prompting aberrant cellular processes such as growth, proliferation, survival, migration and loss of tissue-specific differentiation traits. Functionally, the V600E mutation in the *BRAF* gene causes an increase in BRAF kinase activity, compared to the wild type form, and constitutively triggers the downstream MEK/ERK pathway independently from extracellular stimuli [22,33]. Nevertheless, the biological impact of BRAF^V600E^ mutation is contingent on cancer type in which the genetic alteration occurs. 

## 3. Role of BRAF^V600E^ Mutation in Thyroid Cancer

In the context of TCs, the BRAF^V600E^ genotype occurs in about 29–83% of cases [15], being more frequent in PTCs and anaplastic/poorly differentiated PTC-derived tumors compared to other histological subtypes, such as FTC and MTC. It should be noted that both activating mutations in *RAS* and *BRAF* genes and RET/PTC rearrangements are mutually exclusive in PTCs, this suggesting that each one might be sufficient for the malignant transformation of thyroid cells. In a clinical perspective, the BRAF^V600E^ genotype in PTCs is generally associated with aggressive clinical phenotypes [34,35], higher rates of disease recurrence [36] and shorter disease-free and OS [37], compared to BRAF wild-type TCs. Nevertheless, this evidence is still matter of debate since more recent studies evidenced that BRAF mutation cannot be considered as independent poor-outcome prognostic factor [38,39], but it should be evaluated in association with other prognostic variables. Likely, these conflicting results may depend on some limitations in experimental study design, such as the methodology for prognostic value of BRAF^V600E^ mutation, enrolling criteria and group size, time of the follow-up, statistical analysis methods, techniques for BRAF^V600E^ detection and result validation. The prognostic significance of BRAF^V600E^ in poorly differentiated/anaplastic TCs harboring this mutation (20–50% of cases) is still unclear [40], probably due to the complexity of genomic alterations in ATCs [41]. 

The biological impact of the BRAF^V600E^ mutation in thyroid tumorigenesis is well characterized. As known, BRAF^V600E^-positive PTCs show the constitutive activated RAF/ERK pathway with downstream repression of many thyroid-specific genes, leading to cell de-differentiation, tumor progression and acquisition of more aggressive phenotypes. Among genes down-regulated by the constitutive activation of BRAF pathway, there are several tumor suppressor genes involved in numerous cellular processes, such as metallopeptidase inhibitor 3 (*TIMP3*), sodium-coupled monocarboxylate transporter 1 (*SLC5A8*), death-associated protein kinase (*DAPK*), b2 retinoic acid receptor (*RARb2*) and their silencing by promoter hypermethylation represents an important molecular mechanism in tumor progression [42]. Moreover, the BRAF^V600E^ mutation has been significantly correlated with the over-expression of matrix-metalloproteases (MMPs), such as MMP-2, MMP-3, MMP-9 and MMP-13 involved in extracellular-matrix degradation and thus tumor invasiveness [43].

A notable consequence of the effect played by the BRAF^V600E^ mutation on TC differentiation is the severe alteration in iodine absorption and accumulation by cancer cells. Under physiological conditions, iodine is transported into thyrocytes by the sodium-iodide symporter (NIS) and incorporated in thyroid hormones [44]. In particular, the first step of thyroid hormone synthesis is the pendrin-mediated efflux of iodide across the apical membrane. Once into the follicle, iodide is oxidized and organified by thyroid peroxidase (TPO) and finally incorporated into thyroglobulin (Tg) [44]. The gain-of-function BRAF^V600E^ mutation has a direct control on the expression of NIS gene (*SLC5A5*) and several other genes involved in iodine metabolism, namely pendrin (*AIT*), *TPO*, *Tg* and thyroid-stimulating receptor (*TSHR*) [45,46]. Indeed, the BRAF^V600E^-activated ERK pathway regulates histone deacetylation at NIS promoter by controlling histone deacetylase (HDAC) activity and epigenetically silences NIS expression [47]. Other studies demonstrated that the promoter methylation of several other iodine-metabolizing genes plays a role in the dysregulation of iodine metabolism in BRAF-mutated PTCs [42]. Clinically, the impairment in iodine metabolism is the main cause of the poor response of BRAF^V600E^ TCs to RAI therapy. Consistently with poor response to standard therapies, a strong correlation was observed between the presence of the BRAF^V600E^ mutation and traits of tumor aggressiveness, such as as extrathyroid extension, advanced tumor stage at diagnosis, lymph nodes or distant metastases and poor outcome for patients [48,49,50].

## 4. BRAF Inhibitors in Thyroid Cancer Treatment and Mechanisms of Resistance

Early stage TCs are treated with total thyroidectomy and RAI adjuvant treatment, in order to eliminate the remaining thyroid tissue and/or residual neoplastic cells after surgery [51]. Instead, RAI treatment represents the most important therapeutic option for patients with metastatic disease, with a relevant impact on OS [51]. As previously discussed, patients with BRAF^V600E^ TCs are poor responders or refractory to RAI therapy, because this gain-of-function mutation modulates iodine metabolism [42,44,47], this resulting in decreased ability of neoplastic cells to uptake and incorporate radioiodine.

Until a few years ago, the guidelines identified conventional therapies, such as external beam radiotherapy and chemotherapy, as unique alternative treatments for patients with RAI-resistant advanced thyroid cancer. Nevertheless, these approaches showed poor effectiveness playing a mainly palliative rather than therapeutic role. Thus, novel therapeutic strategies for RAI-resistant advanced TCs represent an urgent clinical need.

In light of recent evidence showing a clinical activity of BRAFi in treatment of BRAF^V600E^ melanoma patients, over the last years, BRAFi-based therapies have been proposed for the management of this setting of TCs patients. The first targeted agents approved from the FDA and EMA were sorafenib (Nexavar) and lenvatinib (Lenvima), due to their efficacy in first-line setting for locally advanced or metastatic differentiated thyroid cancer resistant to RAI or other treatments. It is important to note that both sorafenib or lenvatinib are multi-kinase inhibitors, with an inhibitory activity respect to BRAF tyrosine kinase. Indeed, sorafenib blocks several signaling pathways, such as RAS/BRAF/MEK/ERK, vascular endothelial growth factor receptors (VEGFRs), RET/PET and platelet derived growth factor receptor β (PDGFRβ) [52], are all implicated in the pathogenesis of thyroid cancer. As a multi-kinase inhibitor, sorafenib potentially inhibits tumor growth, progression, metastasis and angiogenesis, as well as suppressing anti-apoptotic mechanisms of tumor cells. Its inhibitory activity was demonstrated on both wild-type and BRAF^V600E^-mutant TCs with a significantly longer progression-free survival in patients harboring the BRAF^V600E^ mutation [53]. Despite the significant improvement of progression free survival (PFS), in treated patients over placebo, advantages in terms of OS between the study groups were not highlighted. Thus, based on its poor specificity, sorafenib is considered a first generation BRAF^V600E^ inhibitor. Lenvatinib is another multi-targeted tyrosine kinase inhibitor (TKI), initially developed to inhibit *VEFGRs* (*VEGFR 1*, *2* and *3*), Fibroblast Growth Factor Receptors 1–4 (*FGFR 1–4*), *PDGFRα*, *RET* and v-kit Hardy-Zuckerman 4 feline sarcoma viral oncogene homolog (*KIT*) proto-oncogenes [54]. Additionally, it demonstrated a satisfying efficacy in patients affected by RAS- or BRAF-mutant thyroid cancer [54]. Clinical trials demonstrated benefit more in terms of PFS than in OS, and in 2015, the FDA and EMA approved lenvatinib for the treatment of RAI-refractory DTC (differentiated thyroid cancer) because of its better safety and efficacy [55] compared to other therapies in use. Thus, at present, sorafenib and lenvatinib are two established therapeutic options in this setting, even though the lack of specificity and the toxicity profile still represent relevant clinical issues.

In the perspective to improve personalized therapies and precision medicine in BRAF-mutated TCs, the next step has been the clinical development of small inhibitors that specifically target BRAF^V600E^-mutated kinase. Recently, the FDA approved two small BRAF-specific inhibitors: vemurafenib (Zelboraf) for BRAF^V600E^-positive advanced RAI-refractory thyroid cancer and dabrafenib (Tafinlar) for BRAF^V600E^-mutated metastatic PTC. Vemurafenib blocks downstream processes of MAPK signaling activation, competing with ATP for the binding domain of BRAF^V600E^-mutated monomer, with a lack of activity against wild-type BRAF kinase [56,57]. Similarly, dabrafenib is a reversible and potent ATP-competitive molecule that selectively inhibits the activity of BRAF^V600E^-, as well as BRAF^V600K^-mutated monomers [58]. Noteworthy, preclinical evidence suggest that BRAF inhibitory agents restore RAI uptake in BRAF^V600E^ iodine-refractory thyroid cancer cells, probably reactivating the expression of thyroid-specific genes involved in iodine metabolism [59]. 

Clinical trials evaluated both vemurafenib and dabrafenib as single agents in recurrent or metastatic PTCs refractory to radioactive iodine and positive for the BRAF^V600E^ mutation [60,61]. The clinical benefit of vemurafenib was highlighted by a long PFS (18.2 months) and the achievement of stable disease for at least six months in 35% of patients [60]. Dabrafenib achieved partial responses and stable disease in, respectively, 29% and 45% of patients; the median PFS was 11.3 months and 50% of cases showed a lack of progression for the entire study duration [61]. Compared to vemurafenib, dabrafenib exhibited a better toxicity profile; its lowered toxicity prevented dose reduction [62], even in long-term therapy, and increased the life quality during treatment, a crucial factor in the management of patients with poor clinical outcome. However, the EMA approval of vemurafenib and dabrafenib is still restricted to BRAF^V600E^-mutated melanoma, since their clinical benefit in thyroid cancer patients was not considered reasonable compared to the toxicity profile [60]. Taking advantage of the potential benefit of the differentiating activity of BRAF targeting agents and their potential synergism with RAI treatment, dabrafenib was tested as boost therapy before thyrotropin α-stimulated iodine-131 whole body scan in 10 patients with BRAF V600E-mutant iodine-refractory PTC. Of note, dabrafenib reversed the insensitivity to RAI treatment in DTC patients, re-inducing new radioiodine uptake in six patients (60%); two patients achieved partial responses and four patients stable disease on standard radiographic restaging at three months [63].

Although BRAF^V600E^ inhibitors demonstrated promising clinical results for RAI-refractory and metastatic or recurrent thyroid cancer, a major limitation of this study is the limited number of patients enrolled and the lack of comparison with other more established therapies. Furthermore, lack of or poor clinical response is a frequent event, due to primary or acquired drug resistance. The clinical response to an exclusive BRAF^V600E^-inhibitor-based therapy is frequently transient because paradoxically multiple mechanisms are able to trigger RAS-ERK signaling, leading to the reactivation of cell proliferation. In such a context, primary or intrinsic resistance is defined by lack of clinical benefit after treatment administration, whereas secondary or acquired resistance by the occurrence of progressive disease after an initial clinical response.

One of the causes of primary resistance to pharmacologic therapies is genomic instability. For example, it was demonstrated that BRAF^V600E^-mutated PTC cells harbor a copy number gain of myeloid cell leukemia 1, chromosome 1q gene (*MCL1*) and a loss of the tumor suppressor cyclin-dependent kinase inhibitor 2A (*CDKN2A*), which confer intrinsic resistance to vemurafenib treatment due to impairment of the B-Cell CLL/Lymphoma 2 (BCL2)-regulated apoptotic pathway [64]. Indeed, these genomic abnormalities favor cancer cell survival and offer the escape from the cell-cycle arrest induced by BRAFi. Consistently, the combination of obatoclax (BCL2/MCL1 inhibitor) with vemurafenib enhances BRAFi activity due to the pro-apoptotic effect of the combinatorial inhibition in vemurafenib-resistant cells [64]. The PIK3CA^H1047R^ activating mutation, common in TC, takes part in TC dedifferentiation process, promoting PTC progression to ATC. In TCs harboring BRAF^V600E^ mutation, the mutated form of phosphatidylinositol-4,5-bisphosphate 3-kinase catalytic subunit alpha (PI3KCA) paradoxically hyperactivates ERK signaling, thus conferring resistance to BRAFi and sustaining tumor progression [65].

Several studies reported that a long-term exposure to vemurafenib induces resistance to BRAF^V600E^ inhibitors, caused by new mutational events or clonal expansion of tumor cells with preexisting resistance mutations. Under BRAFi pressure, PTCs cells develop the KRAS^G12D^ mutation, leading to a constitutively active form of this GTPase, insensitive to the regulation of GTPase-activating proteins [66]. This mutation confers adaptive resistance to vemurafenib because it overcomes MAPK-signaling inhibition through receptor tyrosine kinase-mediated activation of PI3K/AKT pathway [66]. In a case report of a patient with RAI-refractory metastatic PTC exposed to a prolonged treatment with vemurafenib, the genetic analysis revealed an unusual spontaneous mutation in *NRAS* gene (NRAS^Q61K^) responsible for acquired resistance to vemurafenib [67]. De novo mutations in the RBM (RNA-binding motifs) gene family (i.e., *RBMX*, *RBM10*) and the consequent amplification of chromosome 5 are associated with secondary resistance to vemurafenib because of the crucial role of RBM proteins in cell-cycle checkpoint regulation and chromosomal segregation [68].

Autocrine signals has been found responsible for secondary resistance to BRAFi based on the evidence that drug-resistant cells may induce autocrine loops to reactivate ERK signaling and overcome BRAF pharmacological inhibition (Table 1). This scenario opens to combined inhibition of BRAF and autocrine pathway of drug resistance to improve patients’ outcome. 

In undifferentiated TC cells treated with vemurafenib, it was observed an autocrine secretion of neuregulin-1 (NRG1), with consequent induction of human epidermal growth factor receptor 3 (HER3)-ligand-dependent activation of HER2/HER3 signaling pathway [69]. Under MAPK signaling suppression, the delocalization of C-terminal-binding proteins (CtBPs) from the *HER3* gene promoter is frequently observed, leading to *HER3* over-expression and phosphorylation. In order to block this mechanism of drug resistance, the combined treatment with lapatinib, an HER receptor inhibitor able to prevent ERK phosphorylation and BRAF (vemurafenib) or MEK (AZD6244) inhibitors has been proposed. The synergistic effect of the combined HER and BRAF blockade increases the sensitivity of TC cells to the single treatment, as confirmed by the inhibition of mitotic cell rates and the significant reduction of thyroid volume in BRAF^V600E^-mutated mice [69]. Similarly, Byeon et al., suggested another autocrine loop involved in re-activation of MAPK and PI3K/AKT pathways in ATC and PTC cell lines under BRAF^V600E^ pharmacological inhibition. BRAFi cause overexpression and autocrine activation of c-Met receptor, which sustains ERK signaling cascade rebound. The combination of vemurafenib with the c-Met inhibitor PHA665752 resulted in the suppression of both AKT and ERK phosphorylation and enhanced therapeutic activity [70]. Moreover, BRAFi-resistant TC cell lines are able to upregulate and secrete interleukine 6 (IL6) under conditions of MAPK pathway inhibition. IL6 is an activator of STAT3/JAK signaling and its autocrine release might support resistance to MAPK pathway inhibition [80]. Indeed, STAT3 pathway is upregulated in response to vemurafenib in vitro, with maximal induction after short-term treatments. The simultaneous blockade of STAT3 and BRAF is able to enhance the sensitivity of TC cells to BRAF^V600E^ inhibitor as well as the combined exposure to tocilizumab (humanized anti-human IL6 receptor antibody) and vemurafenib increases the activity of BRAFi single agent [71], confirming the crucial role of IL6/STAT3 axis in modulating the sensitivity of TC cells to BRAFi.

The feedback activation of the EGFR signaling pathway is a mechanism responsible for the attenuation of the response to vemurafenib as a single agent in TC cells and in other BRAF-mutated human malignancies. Indeed, EGFR is commonly overexpressed in TCs, especially in PDTCs [81]; however, the response of TC cells to the EGFR inhibitor, gefitinb, is negligible. Recent evidence suggests that BRAF^V600E^ inhibition stimulates EGFR phosphorylation and consequently ERK- and AKT-signaling reactivation. Our group recently demonstrated that the simultaneous inhibition of EGFR and BRAF pathways may represent a strategy to potentiate BRAFi single agent [72]. Noteworthy, this observation is consistent with other reports suggesting that EGFR signaling rebound activation is likely a mechanism responsible for resistance to vemurafenib in cancer cell models characterized by high EGFR expression, such as colorectal cancer cells. By contrast, the rebound activation of EGFR signaling is absent in melanoma cells that are characterized by poor EGFR expression [82]. 

A recent study evaluated the role of the mTOR (mammalian target of rapamycin) pathway in resistance to vemurafenib in thyroid cancer. It was observed that the combined administration of vemurafenib and mTOR inhibitors, such as metformin and rapamycin, decreased cell viability and increased cell death in thyroid cancer cell lines harboring the BRAF^V600E^ mutation [74].

Due to the promising results obtained by synergistic inhibition of different pathways in preclinical models, at present, the research is focused on combination therapies in order to prevent or by-pass resistance mechanisms. In 2018, the FDA approved the first combination treatment with the BRAF^V600E^ inhibitor dabrafenib and the MEK inhibitor trametinib (Mekinist) for the management of unresectable or metastatic BRAF^V600E^-positive ATC. Even if the number of patients with eligibility criteria of the multicenter nonrandomized trial was small, the drug combination efficacy was clearly demonstrated: 57% of patients achieved a partial response and 4% a complete response. Among responding patients, 64% showed stable disease for at least six months [40]. However, recently, it was demonstrated that the clinical activity of the combined BRAF/MEK inhibition is partially impaired by KRAS acquired mutations which by-pass the dual-blockade [83].

In order to overcome both primary and/or acquired resistance in unresectable and/or metastatic RAI-refractory thyroid cancer and potentiate the therapeutic activity of BRAF^V600E^ targeted therapy, other anti-cancer drugs or clinical treatments are under investigation as potentially synergic with BRAF^V600E^ inhibitors. Beyond the above-mentioned combined pharmacological strategies, other studies with combination of BRAF^V600E^ inhibitors and anti-cancer agents are under evaluation, even though they are still in pre-clinical phase (Table 1). The aim of drug combinations in patients harboring BRAF mutations is to hinder tumor growth by using agents able to block specific pathways involved in acquired BRAF^V600E^ inhibitor resistance. Vemurafenib and dabrafenib were tested in association with inhibitors of MEK/ERK cascade [75,76], EGFR signaling [73], the pro-survival pathway NF-κB [77], PI3K/mTOR pathways [79] and BCL-2 [78] (Table 1).

Intriguingly, the importance of synergistic approaches is confirmed by six clinical trials, actually enrolling, where vemurafenib and dabrafenib are administered in combination with other treatments for BRAF-mutated thyroid cancer (Table 1). Three trials (NCT03244956, NCT03975231, NCT02145143) are focused on the synergistic anti-tumor effect of BRAF^V600E^ inhibitors and radiotherapy treatments (RAI and IMRT—Intensity-Modulated Radiation Therapy). Two other studies (NCT01947023, NCT02456701) involved molecular-targeted agents: BRAFi in combination with the EGFR tyrosine kinase inhibitor lapatinib, which blocks the downstream mTOR signaling (NCT01947023), or the anti-HER3 monoclonal antibody KTN3379, which efficiently inhibits HER3 activity, suppressing PI3K/AKT pathway (NCT02456701). Finally, one trial (NCT03181100) examined the potentiality of BRAF^V600E^ inhibitor vemurafenib as a single agent or in combination with the MEK inhibitor cobimetinib, and the immune checkpoint inhibitor atezolizumab, which activates the anti-tumor T-cell mediated immune response blocking the interaction between programmed death-ligand 1 (PD-L1) and programmed cell death protein 1 (PD-1) or CD80 receptors (B7-1Rs). Thus, the promising preclinical results of combined therapy with BRAF^V600E^ kinase inhibitors and the numerousness of other targeted agents reinforces the argument of personalized approaches to treatment in TCs.

## 5. Conclusions

BRAF^V600E^ is the most common molecular abnormality in thyroid cancer. This mutation is frequently associated with tumor aggressiveness and poor prognosis because of the constitutive activation of downstream MAPK pathway, which drives cellular dedifferentiation and cancer progression. Clinically, BRAF^V600E^-mutated thyroid cancer cells exhibit primary resistance or poor response to RAI therapy due to the inhibition of radioidine uptake and/or remodeling of metabolic pathways mediated by hyperactive BRAF^V600E^ kinase. Thus, the advent of molecular and genetic screening opened the door to molecular/prognostic characterization of TCs and led the researchers to focus on identification of small molecules, which target BRAF-mutated monomers in a very specific manner. In the recent years, some BRAF^V600E^-targeting agents were approved for the treatment of RAI-resistant BRAF-mutated locally advance or metastatic thyroid cancer.

However, even though these achievements represent major breakthroughs in this setting of patients, several issues are still unsolved and the clinical use of BRAF targeting agents in these patients needs to be further corroborated by new evidence. It should be noted that clinical data, which allowed the approval of BRAFi in TC, were obtained mostly in phase 2 trials based on a limited numbers of patients, which represents a major limit for the wide use of these agents. Furthermore, besides the achievement of interesting rates of objective responses upon vemurafenib or dabrafenib single agent therapy, numerous thyroid cancer patients display rapid disease progression due to primary or acquired drug resistance. 

Drug resistance might occur due to either the expansion of existing resistant clones, harboring intrinsic mutations or the occurrence of new genetic or epigenetic alterations, which often involve signaling molecules up/downstream the pathway targeted by the anticancer agent. In both cases, tumor cells become able to survive in the new microenvironment generated by the anticancer agent. As extensively described above, a number of different mechanisms has been identified at the bottom of BRAFi resistance in thyroid tumor cells, which induce alternative pathways with the final effect to reactivate MAPK cascade. This preclinical evidence opened the scenario to drug-combination approaches as novel strategies to overcome the single agent-induced clonal selection of resistant tumor cells, as well as to stimulate the immune response. However, it should be emphasized that combinations of vemurafenib with other anti-cancer molecules were tested mostly in preclinical models and the only therapeutic combination that was tested clinically is the dual inhibition of BRAF (dabrafenib) and MEK (trametinib), which was evaluated in a phase 2 enrolling 16 BRAF V600E-mutated anaplastic thyroid carcinomas [40]. While this is an interesting result achieved in an orphan disease, these data need to be further validated in the wider setting of BRAF-mutated PTC. Additionally, the possibility of combining BRAFi with other anti-cancer agents is under evaluation in several clinical trials that will eventually achieve two major objectives: (i) to obtain synergistic effects and enhance patients’ sensitivity to BRAFi agents and (ii) to reduce doses of anticancer drugs to limit AEs and improve the quality of life of thyroid cancer patients. In such a context, the possibility to restore sensitivity to RAI therapy upon BRAF inhibition represents an exciting perspective for these patients due to the high efficacy of RAI in DTCs and its favorable toxicity profile. However, data on efficacy and tolerability of these new therapeutic strategies need to be awaited before drawing any conclusions.

## Figures and Tables

**Table 1:** Novel therapeutic strategies based on the combination of BRAF inhibitors with other targeted agents under pre-clinical and clinical development in thyroid carcinoma.

Pre-Clinical Stage					
Combined Therapy	Thyroid Cancer Subtype	Patient Number	Experimental Phase	Drug Targets	Reference
Vemurafenib + Vorinostat	BRAF^V600E^-mutated and wild-type TC cell lines	n.a.	in vitro	dual inhibition of BRAF^V600E^ + histone deacetylases	[47]
Vemurafenib + Obatoclax	BRAF^V600E^-positive PTC cell lines, animal models	n.a.	in vitro and in vivo	dual inhibition of BRAF^V600E^ + BCL2	[64]
Vemurafenib + Pictilisib	BRAF^V600E^-positive BRAF^V600E^/PIK3CA^H1047R^-mutated ATC cell lines, animal models	n.a.	in vitro and in vivo	dual inhibition of BRAF^V600E^ + PI3K	[65]
Vemurafenib + Palbociclib	BRAF^V600E^-positive TC cell lines	n.a.	in vitro	dual inhibition of BRAF^V600E^ + CDK4/6	[68]
Vemurafenib + Lapatinib	BRAF^V600E^-positive TC cell lines, animal models	n.a.	in vitro and in vivo	dual inhibition of BRAF^V600E^ + HER family receptors	[69]
Vemurafenib + PHA665752	BRAF^V600E^-positive ATC and PTC cell lines, animal models	n.a.	in vitro and in vivo	dual inhibition of BRAF^V600E^ + c-Met	[70]
Vemurafenib + Tocilizumab + HO-3867	BRAF^V600E^-positive TC cell lines and TC tissues	n.a.	in vitro	dual inhibition of BRAF^V600E^ + IL-6 (tocilizumab) or STAT3 (HO-3867)	[71]
Vemurafenib + Gefitinib	BRAF^V600E^-positive PTC and ATC cell lines	n.a.	in vitro	dual inhibition of BRAF^V600E^ + EGFR	[72,73]
Vemurafenib + Rapamycin	BRAF^V600E^-positive PTC and ATC cell lines	n.a.	in vitro	dual inhibition of BRAF^V600E^ + mTOR	[74]
Vemurafenib + PD98059	BRAF^V600E^-positive PTC cell lines	n.a.	in vitro	dual inhibition of BRAF^V600E^ + MEK1/2	[75]
Vemurafenib + Selumetinib	BRAF^V600E^- positive PTC cell lines, animal models	n.a.	in vitro and in vivo	dual inhibition of BRAF^V600E^ + MEK1/2	[76]
Vemurafenib + Bortezomib	BRAF^V600E^-positive PTC and BRAF^wt^ ATC cell lines	n.a.	in vitro	dual inhibition of BRAF^V600E^ + proteasome	[77]
Vemurafenib + Navitoclax	BRAF^V600E^-positive PTC cell lines	n.a.	in vitro	dual inhibition of BRAF^V600E^ + BCL-2/BCL-XL interaction with BIM protein	[78]
**Clinical Trials**					
Dabrafenib + Trametinib + Everolimus	BRAF^V600E^- and PIK3CA^H1047R^-positive ATC patient	1	pre-clinical/clinical	combined inhibition of BRAF^V600E^ + MAPK (trametinib) + mTOR (everolimus)	[79]
Dabrafenib + Trametinib + RAI	RAS/BRAF^V600E^-mutated metastatic RAI-refractory DTC patients	87	Phase II	potentiation of RAI activity by BRAF^V600E^ and MEK inhibition	NCT03244956
Dabrafenib + Trametinib + IMRT	BRAF^V600E^-mutated ATC patients	20	Phase I	potentiation of IMRT activity by BRAF^V600E^/MEK inhibition	NCT03975231
Vemurafenib + RAI	TC patients	12	Pilot study	potentiation of RAI activity by BRAF^V600E^ inhibition	NCT02145143
Dabrafenib + Lapatinib	BRAF^V600E^-mutated patients with unresectable/metastatic TC	21	Phase I	dual inhibition of BRAF^V600E^ + HER family receptors	NCT01947023
Vemurafenib + KTN3379	BRAF-mutant RAI-refractory TC patients	7	Phase I	dual inhibition of BRAF^V600E^ + HER3	NCT02456701
Vemurafenib + Atezolizumab + Cobimetinib	BRAF^V600E^-mutated ATC patients	50	Phase II	combined inhibition of BRAF^V600E^ + PD-L1 (atezolizumab) + MEK (cobimetinib)	NCT03181100

n.a., not applicable.
